# Excellent efficacy of trastuzumab deruxtecan in a patient with HER2-positive advanced breast cancer complicated by pulmonary lymphangitic carcinomatosis: a case report and literature review

**DOI:** 10.3389/fphar.2025.1574286

**Published:** 2025-04-25

**Authors:** Simin Luo, Jie Chai, Linxiaoxiao Ding, Song Tang

**Affiliations:** ^1^ Breast Tumor Center, Sun Yat-sen Memorial Hospital, Sun Yat-sen University, Guangzhou, Guangdong, China; ^2^ The Second Department of General Surgery, The First Affiliated Hospital of Guangdong Pharmaceutical University, Guangzhou, Guangdong, China

**Keywords:** trastuzumab deruxtecan, DS-8201, advanced breast cancer, pulmonary lymphangitic carcinomatosis, case report

## Abstract

Trastuzumab deruxtecan (T-DXd or DS-8201), as a novel antibody–drug conjugate, has demonstrated good efficacy in HER2-positive advanced breast cancer. However, its effectiveness in treating pulmonary lymphangitic carcinomatosis (PLC) has not been previously reported. This paper presents a case of a HER2-positive advanced breast cancer patient who experienced disease progression after treatment with trastuzumab and pertuzumab. The patient developed multiple metastases along with PLC and subsequently received T-DXd treatment, achieving 14.2 months of progression-free survival (PFS). This case is the first to reveal the therapeutic potential of T-DXd in breast cancer patients with PLC.

## Introduction

Breast cancer is the most common cancer type among women (Bray et al., 2024). In clinical practice, breast cancer is classified into different subtypes based on the expression of hormone receptors (HRs), including estrogen receptor (ER) and progesterone receptor (PR), and human epidermal growth factor receptor 2 (HER2) (Waks and Winer, 2019). Among these, HER2-positive breast cancer accounts for approximately 15%–20% of cases (Loibl and Gianni, 2017). Before the advent of HER2-targeted monoclonal antibodies, the prognosis for HER2-positive breast cancer patients was relatively poor (Kunte et al., 2020). The introduction of trastuzumab marked the beginning of a new era in HER2-targeted treatment for breast cancer. In recent years, antibody–drug conjugates (ADCs) have garnered increasing attention. The latest ADC, trastuzumab deruxtecan (T-DXd or DS-8201), has shown remarkable efficacy in patients with HER2-positive advanced breast cancer (Hurvitz et al., 2023; Cortés et al., 2022). T-DXd is composed of an anti-HER2 monoclonal antibody and a topoisomerase I inhibitor (DXd) linked through a cleavable linker, with a drug-to-antibody ratio (DAR) of 8:1, which is much higher than that of traditional ADCs (e.g., T-DM1 has a DAR of 3.5:1), significantly enhancing its cytotoxicity against tumor cells, especially in heterogeneous or low HER2-expressing metastatic lesions (Modi et al., 2020). Additionally, T-DXd has a unique “bystander effect” that allows it to effectively penetrate the tumor microenvironment, making it more advantageous in the treatment of diffuse metastases (Modi et al., 2020; Li et al., 2024). However, there are no published reports on the treatment effect of T-DXd in patients with pulmonary lymphangitic carcinomatosis (PLC), which is a form of diffuse metastasis.

PLC is a rare metastatic pulmonary disease characterized by the diffuse spread of advanced malignant tumors via the pulmonary lymphatics (Klimek, 2019). Breast cancer is the most common primary tumor type associated with PLC, although lung cancer, gastric cancer, and others may also lead to the development of PLC (Klimek, 2019; Ikezoe et al., 1995). Dyspnea and dry cough are the most common symptoms of PLC, occurring in more than half (59.0%) and one-third (33.8%) of patients, respectively (Klimek, 2019). PLC is considered a visceral emergency with a severe prognosis. Approximately half of the patients die within 2 months of the onset of respiratory symptoms and within 3 weeks of hospital admission (Klimek, 2019). Therefore, improving the prognosis for advanced breast cancer patients with concomitant PLC is particularly important.

This case report presents a HER2-positive breast cancer patient who experienced rapid disease progression after treatment with trastuzumab and pertuzumab. After discontinuing treatment for 11 months, the patient developed multiple systemic metastases accompanied by PLC. The patient achieved 14.2 months of progression-free survival (PFS) following T-DXd treatment. This case is the first to reveal the potential therapeutic value of T-DXd in this specific metastatic phenotype.

## Case presentation

### Basic information and initial diagnosis

A 59-year-old female was admitted in August 2021 due to a lump in the right breast. The patient had no significant medical or family history. Breast magnetic resonance imaging (MRI) revealed a mass in the lower quadrant of the right breast, measuring approximately 25 mm × 23 mm × 18 mm, suspected to be breast cancer with multiple right axillary lymph node metastases. Subsequently, the patient underwent fine-needle aspiration biopsy of right breast mass and right axillary lymph nodes, with the following pathological and immunohistochemical results:

① Right breast mass: invasive ductal carcinoma (grade III); ER (−), PR (−), HER2 (3+), and Ki67 (approximately 70%); PD-L1 (22C3) tumor cells <1%; PD-L1 (22C3) immune cells (approximately 20%).
② Right axillary lymph node: metastatic carcinoma; ER (−), PR (−), HER2 (3+), and Ki67 (approximately 40%). Bone single-photon emission computed tomography/computed tomography (SPECT/CT) showed no bone metastasis, and cranial MRI showed no abnormalities. The final diagnosis was right breast invasive ductal carcinoma with right axillary lymph node metastasis, stage III, HER2-positive.

A timeline of the patient’s disease progression and treatment process is shown in Table 1.

**TABLE 1:** Timeline of the patient’s condition and course of treatment.

Date	Incident description
2021-08	After being diagnosed with right breast invasive ductal carcinoma (stage III, HER2-positive), neoadjuvant chemotherapy was initiated (AC regimen: liposomal doxorubicin + cyclophosphamide).
2021-10-26	Right breast modified radical mastectomy with axillary lymph node dissection.
2021-11-23	Postoperative adjuvant therapy included the AC regimen, followed by the sequential THP regimen (albumin-bound paclitaxel + trastuzumab + pertuzumab), and then the sequential HP regimen (trastuzumab + pertuzumab).
2022-04	The patient discontinued adjuvant therapy on their own.
2023-03-24	First-line treatment with T-DXd was initiated after the development of systemic metastasis combined with PLC.
2024-05-30	Radiological imaging confirmed disease progression (PFS 14.2 months).
2024-06-12	Second-line treatment was initiated with capecitabine, pyrotinib, and nimotuzumab.

### Neoadjuvant chemotherapy and surgery

The patient received two cycles of neoadjuvant chemotherapy (AC regimen: liposomal doxorubicin + cyclophosphamide), but the tumor did not significantly shrink, and the patient requested early surgery. On 26 October 2021, the patient underwent right breast modified radical mastectomy and right axillary lymph node dissection. Postoperative pathology revealed chemotherapy-induced changes (Miller–Payne grade: G1), with 13 out of 28 axillary lymph nodes being positive for metastasis. Both the right breast and axillary lymph nodes showed HER2(2+) with positive fluorescence *in situ* hybridization (FISH). The postoperative diagnosis was right breast invasive ductal carcinoma, postoperative ypT2N3M0, stage IIIc, HER2-positive.

### Postoperative adjuvant therapy

From 23 November 2021 to 30 March 2022, the patient received two cycles of the AC regimen, four cycles of the THP regimen (albumin-bound paclitaxel + trastuzumab + pertuzumab), and three cycles of the HP regimen (trastuzumab + pertuzumab). During this period, the patient received radiotherapy at an outside hospital, but the specific regimen is unknown. The patient then discontinued treatment on her own.

### Systemic multiple metastasis of breast cancer with concurrent PLC

In November 2022, the patient noticed enlarged left cervical lymph nodes, but no further treatment was given. In March 2023, the patient developed shortness of breath, cough, orthopnea, and severe headaches. A CT scan performed at an outside hospital revealed multiple lung metastases, T3–4 vertebral and adjacent bone metastases, multiple lymph node metastases in the mediastinum and left axilla, multiple liver metastases, and moderate pleural effusion on the right side. A pleural effusion puncture and drainage were performed, yielding pale-red blood-tinged pleural fluid. Cytology showed atypical glandular carcinoma cells, consistent with metastatic adenocarcinoma. The symptoms of dyspnea and cough improved considerably.

After coming to our hospital, a CT scan was performed, which confirmed the diagnosis of widespread metastasis, including lymph nodes, lungs, pleura, liver, and bones. At the same time, the CT scan revealed thickening of the interlobular septa and nodular enlargement of the bronchovascular bundles in the lower lobes of both lungs. After multidisciplinary consultations with the departments of oncology, radiology, pulmonology, and pathology and a comprehensive assessment of the patient’s history, CT findings, and pleural effusion cytology, infectious causes were excluded, and the pulmonary findings were determined to be consistent with PLC. Cranial MRI showed multiple new nodules in the bilateral temporal lobes, right parietal lobe, and left occipital lobe, considered to be metastatic tumors. The final diagnosis was as follows:

① Postoperative right breast cancer with multiple metastases (lymph nodes, lungs, pleura, liver, brain, and bones), stage IV, HER2-positive.
② PLC.

### T-DXd treatment

**FIGURE 1:**
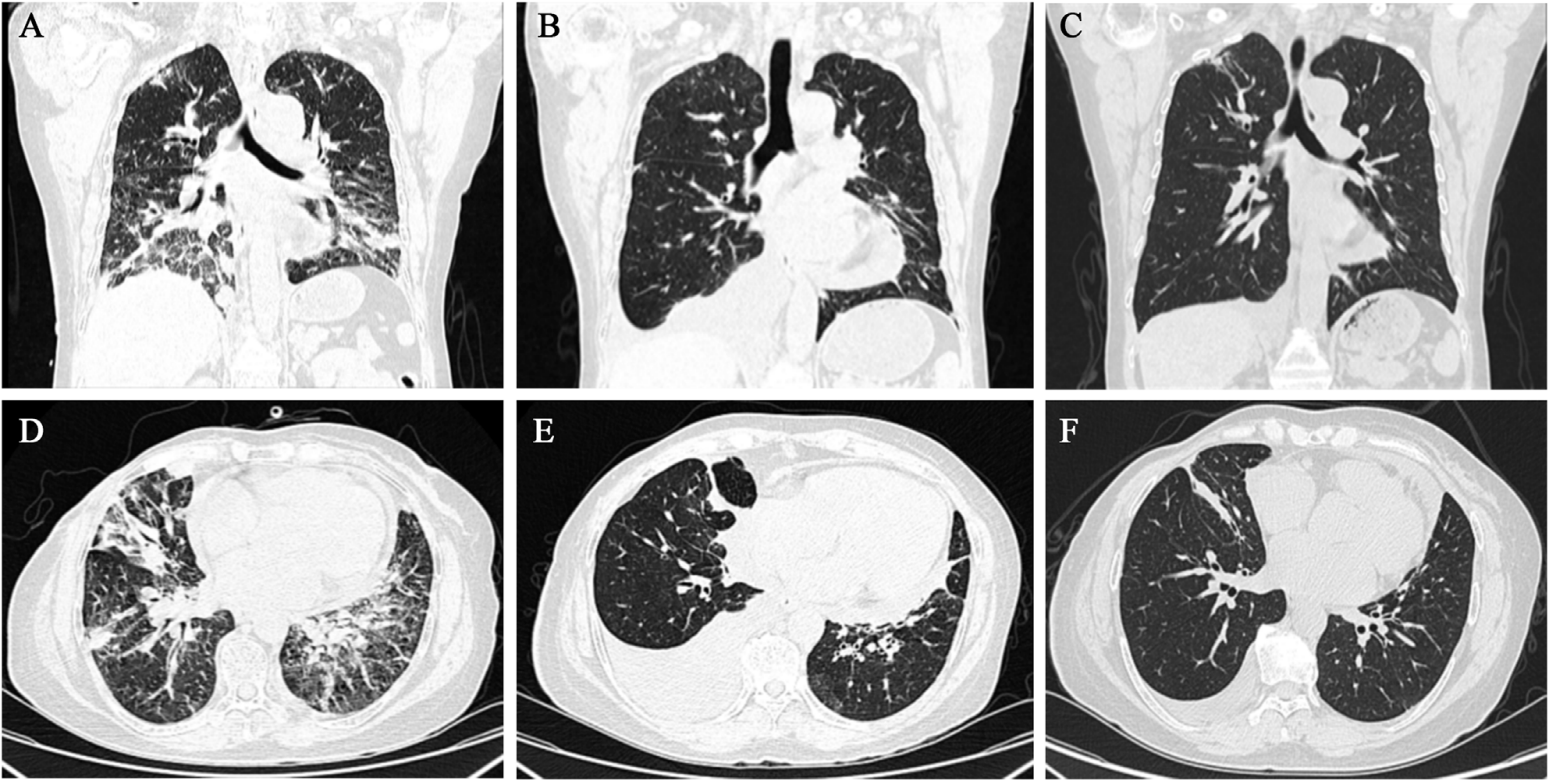
Chest CT showed gradual improvement of PLC following T-DXd treatment. **(A–C)** Cross-sectional images taken on 20 March 2023, 10 July 2023, and 14 November 2023, respectively. **(D–F)** Coronal images taken on 20 March 2023, 10 July 2023, and 14 November 2023, respectively.

**FIGURE 2:**
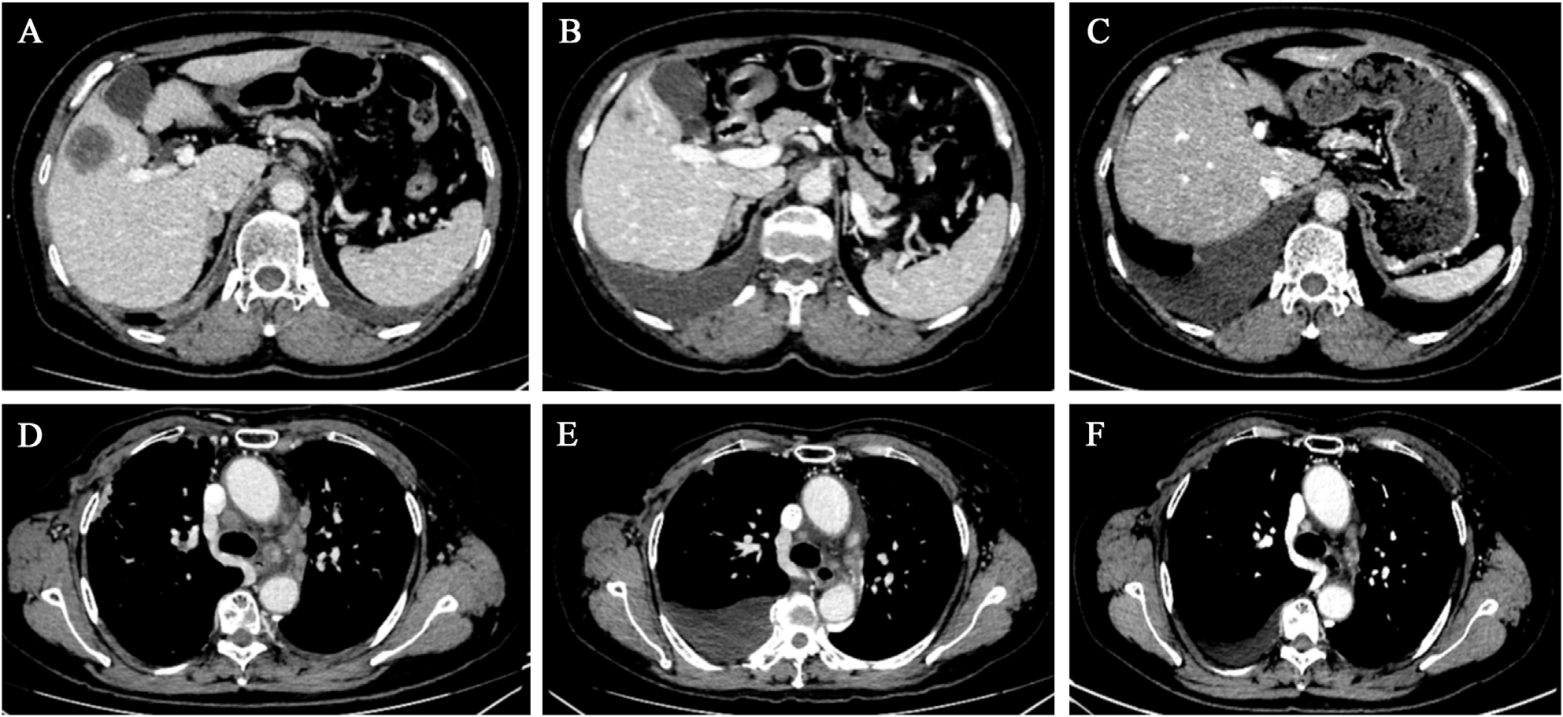
Chest and abdominal CT scans revealed a gradual reduction in the size of the hepatic metastases and mediastinal lymph nodes. **(A–C)** Liver cross-sectional images taken on 20 March 2023, 10 July 2023, and 14 November 2023, respectively. **(D–F)** Mediastinal cross-sectional images taken on 20 March 2023, 10 July 2023, and 14 November 2023, respectively.

**FIGURE 3:**
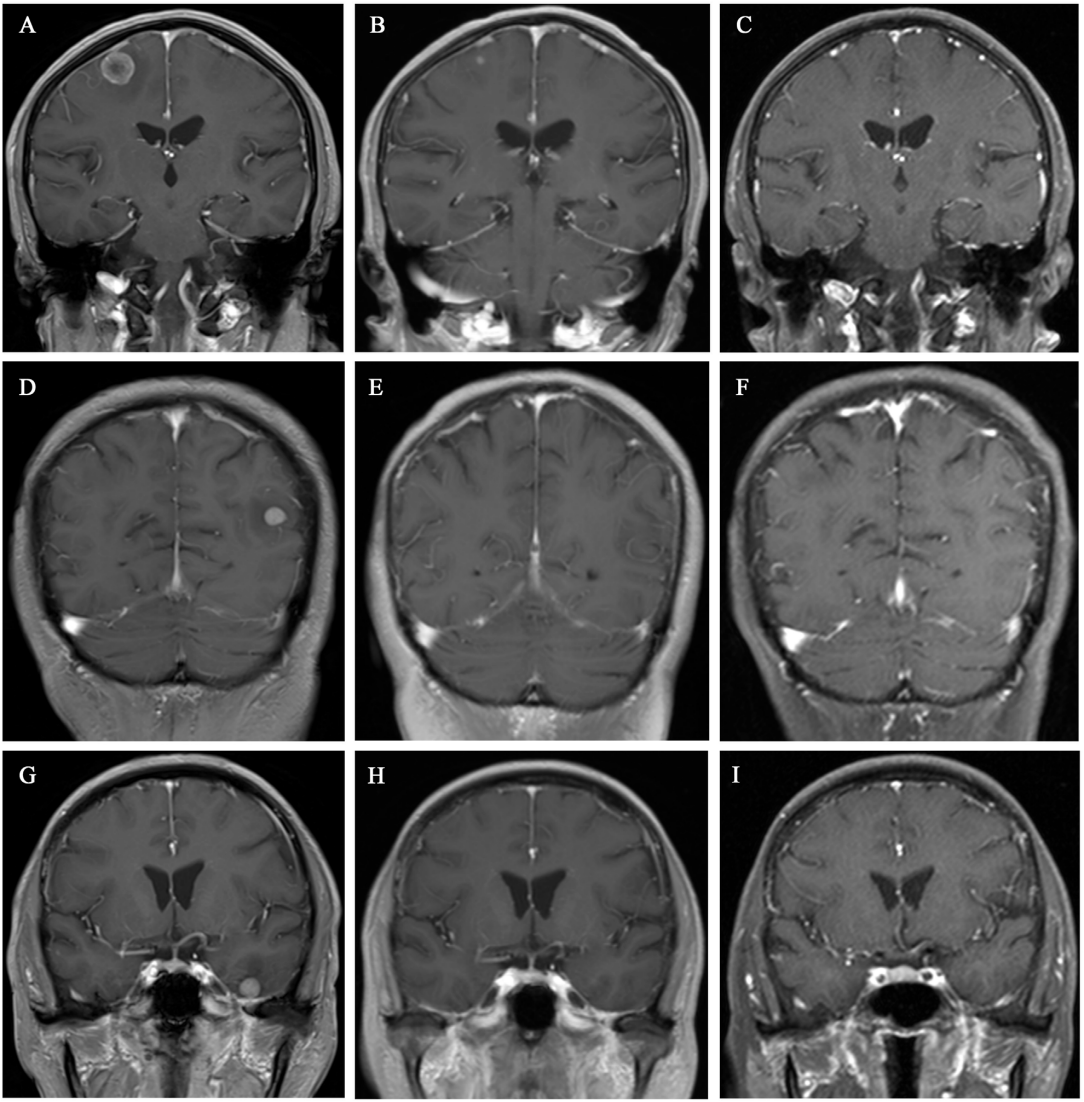
Cranial MRI showed gradual shrinkage of the brain metastases in various regions. **(A–C)** Coronal images of the left parietal lobe taken on 20 March 2023, 10 July 2023, and 14 November 2023, respectively. **(D–F)** Coronal images of the right temporal lobe taken on 20 March 2023, 10 July 2023, and 14 November 2023, respectively. **(G–I)** Coronal images of the right occipital lobe taken on 20 March 2023, 10 July 2023, and 14 November 2023, respectively.

After a multidisciplinary consultation, the patient was considered to have PLC, a visceral emergency, with concurrent intracranial and widespread systemic metastases, resulting in a poor prognosis. First-line treatment options included T-DXd or pyrotinib + capecitabine. The patient chose T-DXd treatment. Treatment began on 24 March 2023, with one infusion every 3 weeks. A total of 18 treatments were completed by 30 May 2024. Follow-up chest and abdominal CT along with cranial MRI revealed new metastatic lesions in the lungs and brain, along with interstitial pneumonia in the lungs (asymptomatic). Despite this, the patient’s PFS had exceeded 14 months. During treatment, the patient’s symptoms of chest tightness, dyspnea, cough, and headaches significantly improved, and her performance status markedly improved. Follow-up chest and abdominal CT (Figures 1, 2) and cranial MRI (Figure 3) also showed a significant reduction in PLC, intracranial metastases, and other systemic organ metastases. Specifically, regarding the pulmonary lesions, before treatment (20 March 2023), there was diffuse interlobular septal thickening in both lower lungs (average thickness 3.5 mm, with a “paving stone” appearance), accompanied by multiple miliary-like nodules (the largest diameter was 5 mm). After treatment (14 November 2023), the interlobular septal thickening significantly decreased (average thickness 1.2 mm), and the number of nodules reduced by 70% (the largest diameter ≤2 mm), meeting the imaging criteria for PLC remission (Klimek, 2019). Regarding the liver metastases, before treatment, the tumor in segment 5 had a maximum diameter of 31 mm, and in segment 8, it was 15 mm. After treatment, the tumor in segment 5 reduced to 8 mm (a 74.2% reduction), and in segment 8, it reduced to 4 mm (a 73.3% reduction), meeting the partial remission criteria, according to RECIST 1.1 (Eisenhauer et al., 2009). Regarding brain metastases, before treatment, there were lesions in the right frontal lobe (15 mm), left temporal lobe (12 mm), and left occipital lobe (10 mm), along with multiple lesions in both temporal lobes. After treatment, the right frontal lobe lesion reduced to 3 mm (an 80.0% reduction), and the lesions in both temporal lobes and the left occipital lobe completely disappeared, meeting the criteria for brain metastasis treatment response (Lin et al., 2015).

### Subsequent antitumor therapy

Due to tumor progression, the patient switched to second-line treatment on 12 June 2024: capecitabine + pyrotinib + nimotuzumab, and by December 2024, the patient continued with this regimen and refused radiotherapy.

## Discussion

Breast cancer is the most common cancer in women worldwide (Bray et al., 2024). HER2 amplification and/or overexpression can be found in 15%–20% of invasive breast cancers and is associated with an aggressive phenotype and poor clinical outcomes (Loibl and Gianni, 2017; Marra et al., 2024). Preclinical studies have shown that HER2 overexpression can upregulate lymphangiogenic factors such as VEGF-C, promoting lymphatic invasion (Su et al., 2006). However, HER2-targeted therapies have changed the biological characteristics of this disease.

Trastuzumab, a monoclonal antibody targeting HER2, was first introduced in clinical trials in the 1990s. Research has demonstrated that the addition of trastuzumab to standard adjuvant chemotherapy significantly improves disease-free survival and overall survival in HER2-positive breast cancer patients (Waks and Winer, 2019). Subsequently, the CLEOPATRA trial established the combination of trastuzumab, pertuzumab, and docetaxel as the standard first-line treatment (Swain et al., 2020). In recent years, ADCs have gained increasing attention. Emactuzumab (T-DM1) was the first ADC approved for breast cancer. The EMILIA trial showed that T-DM1 significantly extended PFS and overall survival in HER2-positive metastatic breast cancer patients who had previously received trastuzumab and taxane treatment (Verma et al., 2012).

With the continued development of the DESTINY Breast series of studies, T-DXd is increasingly used in the clinical practice of advanced breast cancer. The results of the DESTINY-Breast01 trial showed that T-DXd demonstrated exceptional efficacy in patients previously treated with T-DM1, with an objective response rate (ORR) of 60.9%, a disease control rate (DCR) of 97.3%, and a median progression-free survival (mPFS) of 16.4 months (Modi et al., 2020). DESTINY-Breast03 results showed that the mPFS of the T-DXd group was 28.8 months, approximately 4.2 times that of the T-DM1 group, with a 67% reduction in the risk of disease progression or death (Hurvitz et al., 2023). In patients with brain metastases from breast cancer, the ORR of T-DXd was 58.3%, and mPFS was 18.1 months, showing durable efficacy (Jerusalem et al., 2022). However, there have been no reports on the efficacy of T-DXd in patients with advanced breast cancer complicated by PLC.

PLC is a rare metastatic pulmonary disease, with primary tumors commonly being breast cancer, lung cancer, or gastric cancer (Klimek, 2019). However, PLC associated with liver cancer (Zhuang et al., 2014), lip cancer (Babu et al., 2011), head and neck squamous cell carcinoma (Tighe et al., 2014), and thyroid cancer (Fend et al., 1989) is extremely rare. PLC is characterized by the diffuse spread of advanced malignant tumors through pulmonary lymphatic vessels, leading to the accumulation of interstitial fluid and oxygen diffusion impairment, which often results in respiratory dysfunction (Klimek, 2019). The CT features of PLC include interlobular septal thickening, bronchovascular bundle enlargement, and subpleural nodules, which need to be differentiated from interstitial lung diseases (such as nonspecific interstitial pneumonia) (Antoniou et al., 2014). Previous reviews have emphasized that combining the history of the primary tumor and pleural effusion cytology can improve the diagnostic specificity of PLC (Klimek, 2019). In this case, other pulmonary diseases were excluded through multidisciplinary discussion, ensuring diagnostic accuracy. The most common symptoms of PLC are dyspnea and dry cough, with a study showing that 90% of patients experience dyspnea and 77% have a cough (Cömert et al., 2013). Respiratory symptoms such as cough, dyspnea, and respiratory distress in PLC are typically refractory to antispasmodic treatment (Zhang and Huang, 2006). PLC is a life-threatening visceral crisis, and approximately half of the patients die within 2 months after the onset of respiratory symptoms or within 3 weeks of hospitalization (Klimek, 2019). Therefore, improving the prognosis of patients with advanced breast cancer complicated by PLC is crucial.

In this study, we present a case of a HER2-positive advanced breast cancer patient with PLC who achieved 14.2 months of PFS after treatment with T-DXd. This suggests that T-DXd may have significant therapeutic value in such patients. Previously, a similar patient was treated with T-DM1 and showed no disease progression for 8 months, but the subsequent results were not reported further (Yu et al., 2017).

Originally, the combination of trastuzumab and pertuzumab was the standard first-line treatment for HER2-positive advanced breast cancer, with T-DXd as a second-line treatment option. However, given the visceral crisis of PLC and the fact that patients with respiratory symptoms often have a life expectancy of less than 2 months and since this patient had previously received trastuzumab and pertuzumab, we directly recommended T-DXd and the combination of pyrotinib with capecitabine as first-line treatment options. The patient ultimately chose T-DXd. The combination of pyrotinib and capecitabine has significantly improved PFS in previous studies, with manageable toxicity, and can be considered an alternative treatment option for HER2-positive metastatic breast cancer patients after trastuzumab and chemotherapy (Xu et al., 2021).

Although T-DXd showed good efficacy in treating HER2-positive advanced breast cancer complicated by PLC, the patient also developed asymptomatic interstitial pneumonia as new lung metastases emerged. Interstitial lung disease (ILD) and/or pneumonia are specific adverse drug reactions associated with T-DXd, with an incidence of 12.5% for all grades and 2.2% for grade ≥3 (Li et al., 2024). Once ILD/pneumonia occurs, it should be safely managed by involving a multidisciplinary team and promptly initiating steroid treatment (Swain et al., 2022). The symptoms of interstitial lung disease are similar to those of PLC, with the most common symptoms being dyspnea, cough, chest pain, hypoxemia, and low fever (Swain et al., 2022; Johkoh et al., 2021). For asymptomatic or mild ILD (CTCAE grade 1), it is recommended to continue T-DXd treatment with enhanced imaging monitoring (every 4–6 weeks) rather than stopping the medication immediately (Swain et al., 2022). However, since this patient experienced tumor progression, the treatment regimen was adjusted. Since PLC itself is a type of interstitial lung disease, it is unclear whether its occurrence will exacerbate the development of interstitial pneumonia during treatment, and there is no relevant research on this yet.

## Conclusion

We present the first case of T-DXd treatment in a patient with advanced breast cancer complicated by PLC. Although significant efficacy was achieved, the patient also developed interstitial pneumonia during tumor progression. The efficacy and safety of T-DXd in treating advanced breast cancer with PLC still require further validation through large-scale, multicenter prospective studies. Additionally, the potential association between PLC and ILD, along with appropriate clinical management strategies, needs further exploration.

## Data Availability

The original contributions presented in the study are included in the article/supplementary material; further inquiries can be directed to the corresponding authors.
